# Absorption-Dominant mmWave EMI Shielding Films with Ultralow Reflection using Ferromagnetic Resonance Frequency Tunable M-Type Ferrites

**DOI:** 10.1007/s40820-023-01058-w

**Published:** 2023-03-28

**Authors:** Horim Lee, Seung Han Ryu, Suk Jin Kwon, Jae Ryung Choi, Sang-bok Lee, Byeongjin Park

**Affiliations:** Composites Research Division, Korea Institute of Materials Science, 797 Changwondaero, Seongsan-Gu, Changwon, Gyeongsangnam-Do 51508 Republic of Korea

**Keywords:** 5G communication, MmWave, EMI shielding, M-type ferrites

## Abstract

Although there is a high demand for absorption-dominant electromagnetic interference (EMI) shielding materials for 5G millimeter-wave (mmWave) frequencies, most current shielding materials are based on reflection-dominant conductive materials. While there are few absorption-dominant shielding materials proposed with magnetic materials, their working frequencies are usually limited to under 30 GHz. In this study, a novel multi-band absorption-dominant EMI shielding film with M-type strontium ferrites and a conductive grid is proposed. This film shows ultralow EMI reflection of less than 5% in multiple mmWave frequency bands with sub-millimeter thicknesses, while shielding more than 99.9% of EMI. The ultralow reflection frequency bands are controllable by tuning the ferromagnetic resonance frequency of M-type strontium ferrites and composite layer geometries. Two examples of shielding films with ultralow reflection frequencies, one for 39 and 52 GHz 5G telecommunication bands and the other for 60 and 77 GHz autonomous radar bands, are presented. The remarkably low reflectance and thinness of the proposed films provide an important advancement toward the commercialization of EMI shielding materials for 5G mmWave applications.

**Supplementary Information:**

The online version contains supplementary material available at 10.1007/s40820-023-01058-w.

## Highlights

- A novel multi-band absorption-dominant electromagnetic interference (EMI) shielding film with transition metal-doped M-type strontium ferrites composite layer and a conductive grid is developed.
- This film shows (1) ultralow EMI reflection less than 5%, (2) in multiple mmWave frequency bands corresponding to ferrites and grid characteristics, (3) with a broadband EMI shielding performance over 99.9% from 40 to 90 GHz.

**Supplementary Information:**

The online version contains supplementary material available at 10.1007/s40820-023-01058-w.

## Graphical abstract

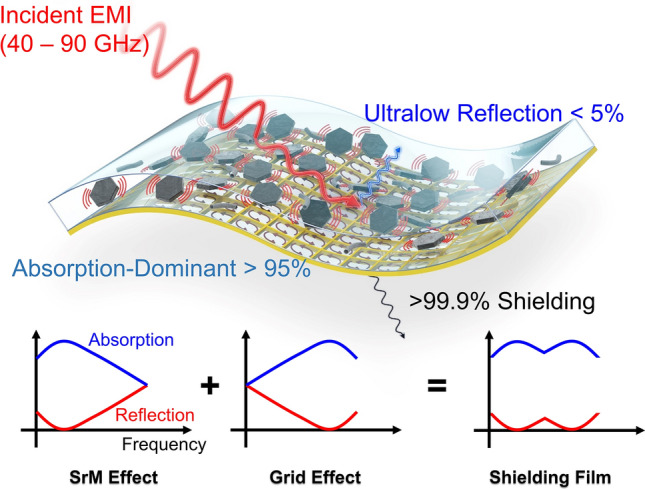

## Introduction

Although current 4G long-term evolution (LTE) telecommunication works in 2.1 GHz frequency bands, 5G telecommunication requires at least 10 times higher frequency bands over 26 GHz, commonly known as millimeter waves (mmWave, 30–100 GHz). Moreover, 5G telecommunication leads to more usage of electronic and telecommunication devices working in high- and multiple-frequency bands, including mobile phones (e.g., 26, 39, and 52 GHz) and autonomous vehicles (e.g., 60 and 77 GHz). This stimulates an increasing concern about electromagnetic interference (EMI) between these devices, which causes electronic malfunctions and even their complete failures [1, 2]. For example, EMI in autonomous vehicle radars may cause misdetection or false detection of targets and lead to severe accidents [3]. Thus, there is a high demand for (1) thin shielding materials, (2) working in mmWave, (3) with broadband/multi-band shielding capability.

EMI shielding materials can be classified into reflection-dominant and absorption-dominant types according to the reflection and absorption contribution to the total shielding [4]. For previous generations of telecommunication, reflection-dominant shielding materials, including metal [2, 5–7], carbon nanotubes [8–12], graphene [13–15] and MXene-based [1, 16–19] materials, have been widely used with their high shielding effectiveness (SE) over 40 dB (99.99% EMI shielding). Due to their high conductivity, they can effectively reflect more than 90% of the external EMI (≥ 10 dB reflection shielding effectiveness (SER)) and minimize its transmission even as a thin film less than 20 μm thickness. However, the reflected EMI may cause secondary radiation pollution, which generates additional superposition and interference with other electromagnetic waves. This is a more serious issue with a short wavelength of 5G mmWave frequency bands and tight component-to-component spacing in integrated 5G mobile modules [20]. Therefore, there is a high demand for absorption-dominant 5G EMI shielding materials with low reflection and high absorption of EMI [21–23]. The SER of absorption-dominant shielding materials should not be higher than 3 dB as it will reflect more than 50% of EMI [24].

To solve this problem, there have been several studies on absorption-dominant shielding materials working at mmWave frequency bands. Although each approach has its own advantages, most of the literature is limited to the 26 GHz 5G band [25–30], and absorption-dominant shielding materials for mmWave over 40 GHz are rarely reported [31, 32, 33, 34]. Foam materials are widely known to be very effective absorption-dominant materials due to their well-matched impedance to air [25, 27, 28, 34]. Zhao et al. reported a 4 mm thick PVDF/multiwall carbon nanotube (MWCNT) foam with 17.1 dB SE and 1.6 dB SER (31% reflection) at 40 GHz [34]. However, due to the high void content of foam materials, their thickness usually needs to be more than several millimeters to achieve satisfactory SE over 20 dB (99% EMI shielding), which limits their application to mobile electronics. Though Ma et al. proposed an interesting multilayer structure with a conductive film and a porous foam to achieve a high SE of 32.6 dB and ultralow SER of 3.1 × 10^–4^ dB (0.7% reflection) with reduced material thickness, its thickness is still 1.95 mm for Ku-Band (12–18 GHz) applications [35]. Magnetic material-based composites have also been of interest due to their low conductivity and high magnetic loss [26, 36]. There have been various attempts to increase the magnetic loss of magnetic materials including one-dimensional magnetic chain [37–39], core–shell structures [40–43], and regulated structural defects [44]. However, as most common magnetic materials, e.g., Fe or Co, lose their magnetic characteristics over 30 GHz, their EMI shielding performance is usually unsatisfactory in mmWave frequency bands. Zhang et al. proposed an AlCoCrFeNi alloy composite working at 40 GHz with a 1 dB SER (20% reflection) and 2 mm thickness; however, its SE was limited to 15 dB [31]. Although the authors’ group attempted to improve the SE over 20 dB and minimize the SER below 0.5 dB (10% reflection) at 0.4 mm thickness with Fe composites and conductive grids, these applications are still limited to 26 GHz [29, 30].

Recently, M-type ferrite has been widely investigated as an effective EMI shielding material for mmWave frequency bands [45, 46]. Ferromagnetic materials are known to exhibit high magnetic loss at a specific frequency band via ferromagnetic resonance (FMR). While most soft magnetic materials (e.g., Fe and Co) and ferrites (e.g., spinel, garnet, W-, X-, Y-, and Z-type) have a low FMR frequency below several GHz, M-type ferrite can induce FMR at over 45 GHz due to its higher magnetic anisotropy than other magnetic materials [47]. Since it is known that the FMR frequency of M-type ferrite is proportional to the magnitude of magnetic anisotropy, the concept of changing its magnetic anisotropy to control the FMR frequency band has received much attention from many researchers [48]. In particular, the substitution of Fe^3+^ ions constituting M-type ferrite into various transition metal ions is considered an effective method to change the magnetic properties of M-type ferrite [49–60]. For example, Co^2+^Ti^4+^ and Al^3+^ are known to be effective elements to decrease and increase magnetic anisotropy, respectively [59, 61]. However, there has been few reports on relating the effect of ion substitution, magnetic anisotropy change, and the FMR frequency shift in mmWave frequency bands [52–55]. In addition, as FMR occurs at a specific frequency band, it is difficult to achieve broadband or multi-band EMI shielding performance using M-type ferrites only.

**Fig. 1:**
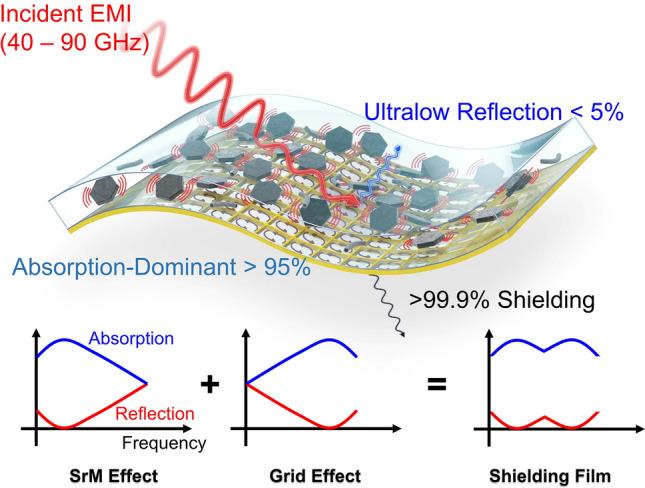
Schematic diagram of the proposed shielding film with a transition metal-doped M-type strontium ferrites composite layer and a conductive grid

In this study, a novel multi-band absorption-dominant EMI shielding film with transition metal-doped M-type strontium ferrites (SrMs) is proposed (Fig. 1). This film shows (1) ultralow EMI reflection less than 5% (0.1 dB SER), (2) in multiple mmWave frequency bands, (3) with a broadband EMI shielding performance over 30 dB SE (99.9% EMI shielding) from 40 to 90 GHz. By integrating a SrM composite layer and a conductive Cu grid, ultralow SER and high SE are simultaneously achieved with sub-millimeter thicknesses. The ultralow reflection frequency bands are controllable by tuning the FMR frequency of the SrM and composite layer dimensions, and a transmission line model is proposed for the theoretical design of the shielding films. Two examples of the proposed shielding films are presented: one for 39 and 52 GHz 5G telecommunication bands and the other for 60 and 77 GHz autonomous radar bands. For both applications, the shielding films show less than 5% ultralow EMI reflection at the desired multiple frequency bands while shielding 99.9% EMI with 0.58 and 0.34 mm thicknesses, respectively. To the best of the authors’ knowledge, this is the first reported absorption-dominant mmWave shielding material for multiple frequency bands.

This paper is organized as follows. First, a FMR frequency tunable SrM is introduced, and its magnetic characteristics are discussed. Second, the design factors for the proposed EMI shielding films are explained with a transmission line model. Then, two examples of shielding films are presented with measured a SE and compared with transmission line model predictions. The shielding performance of the proposed shielding films is also compared with previously reported literature.

## Experimental

### Materials

The precursors used for doped SrMs were strontium nitrate (Sr(NO_3_)_2_), iron nitrate nonahydrate (Fe(NO_3_)_3_·9H_2_O), cobalt nitrate hexahydrate (Co(NO_3_)_2_·6H_2_O), titanium isopropoxide (Ti(OCH(CH_3_)_2_)_4_), aluminum nitrate nonahydrate (Al(NO_3_)_3_·9H_2_O) and citric acid monohydrate (C_6_H_8_O_7_·H_2_O). All the chemicals were purchased from Sigma‒Aldrich and used as received without any further purification. A thermoplastic polyurethane (TPU) solution, 30 wt% TPU dissolved in n,n-dimethylformamide (DMF), from Songwon Industrial, Korea, was used as a polymer binder for the magnetic composite layer. Single-walled carbon nanotube (CNT, JENO 8A) from JEIO Co., Korea is used to control permittivity of the composite layer. A Cu grid, designed by the authors, was manufactured by Samwon ACT, Korea via electroforming.

### Preparation of the M-type Strontium Ferrites

Co-Ti and Al doped SrMs powders (SrFe_12-2x_Co_x_Ti_x_O_19_ and SrFe_12-x_Al_x_O_19_) were synthesized using the citrate sol–gel method. First, stoichiometric quantities of strontium nitrate, iron nitrate nonahydrate, cobalt nitrate hexahydrate, titanium isopropoxide, aluminum nitrate nonahydrate and citric acid monohydrate were dissolved in deionized water. The concentration of Sr^2+^ ions was 0.05 M, and the molar ratio of Sr^2+^ ion: citric acid monohydrate was 1:1. Then, the mixed solution was heated to 90 °C for 24 h to completely evaporate the water. Afterward, the dried gel was gently hand-ground using a mortar with 40 wt% NaCl and calcined at 1,250 °C for 3 h. Finally, the calcined powder was washed 5 times with deionized water to remove residual NaCl and dried at 80 °C for 24 h.

### Preparation of the EMI Shielding Films

A TPU solution and SrMs were mixed with a weight ratio of TPU:SrMs = 3:7 using a planetary mixer (ARE-310, Thinky) for 5 min at 2,000 rpm. A desired fraction of CNT (0.1–0.5 wt%) was mixed together with SrM and TPU only when a higher permittivity of the composite layer is needed. The mixed composite solution was cast into 100 μm thick layers via bar coating. The casted layers were dried for 30 min at 110 ℃ to evaporate unnecessary DMF solvents in the composite layers. Finally, stacked composite layers, with a Cu grid at the bottom, were pressed at 120 ℃ with 10 MPa for 20 min to prepare a shielding film of desired thickness using a thickness gauge.

### Material Characterizations

The morphologies of doped SrM powders and proposed EMI shielding films were analyzed using a scanning electron microscope (JSM-7001F, JEOL) and a transmission electron microscope (JEM-F200, JEOL). The crystal structures of the doped SrM powder were analyzed by X-ray diffraction (D/Max 2500, RIGAKU) with Cu Ka radiation. The Rietveld refinement of XRD data was performed by Fullprof Suite software package to verify the crystal structural analysis. X-ray photonelectron spectrometer (AXIS SUPRA, Kratos) were used to study the chemical bonding and the valence state. The magnetic hysteresis loops were measured using a vibrating sample magnetometer (EZ9 VSM, Microsense) with an applied field of ± 20 kOe.

To obtain the shielding effectiveness of the products, a vector network analyzer (N5291A, Keysight) and a free space measurement system (FS-110, EMLabs) were used for scattering the parameter measurements. Scattering parameters were measured in four different frequency bands: the R (26.5–40 GHz), Q (33–50 GHz), V (50–75 GHz), and W (75–110 GHz) bands. The samples were prepared by cutting the EMI shielding film into rectangular specimens of 10 × 10 cm^2^. The shielding effectiveness and shielding efficiency were calculated from the measured scattering parameters as follows:

1 $$R={\left|{S}_{11}\right|}^{2}$$

2 $$T={\left|{S}_{21}\right|}^{2}$$

3 $$A=1-R-T$$

4 $$\mathrm{SER}=-10\mathrm{log}(1-R)$$

5 $$\mathrm{SEA}=-10\mathrm{log}(T/(1-R))$$

6 $$\mathrm{SE}= \mathrm{SER}+\mathrm{SEA}$$

where S_11_ and S_21_ refer to the scattering parameters; SER, SEA, and SET are the shielding effectiveness of relfection, absorption, and total; and *R*, *T*, and *A* refer to the reflectance, transmittance, and absorbance, respectively, which are also known as shielding efficiency. *R* and *A* can be also calculated from SE as follows, from Eqs. (4) and (5):

7 $$R=\left(1-{10}^{-\frac{\mathrm{SER}}{10}}\right)$$

8 $$A=\left(1-{10}^{-\frac{\mathrm{SEA}}{10}}\right)\times \left(1-R\right)$$

The electromagnetic parameters (complex permeability and permittivity) of the composite layer were also obtained using the aforementioned measurement system. These parameters are calculated from the measured scattering parameters using the Nicholson–Ross–Weir method [62].

## Ferromagnetic Resonance Frequency Tunable M-type Strontium Ferrites

### Microstructure of Doped M-type Strontium Ferrites

**Fig. 2:**
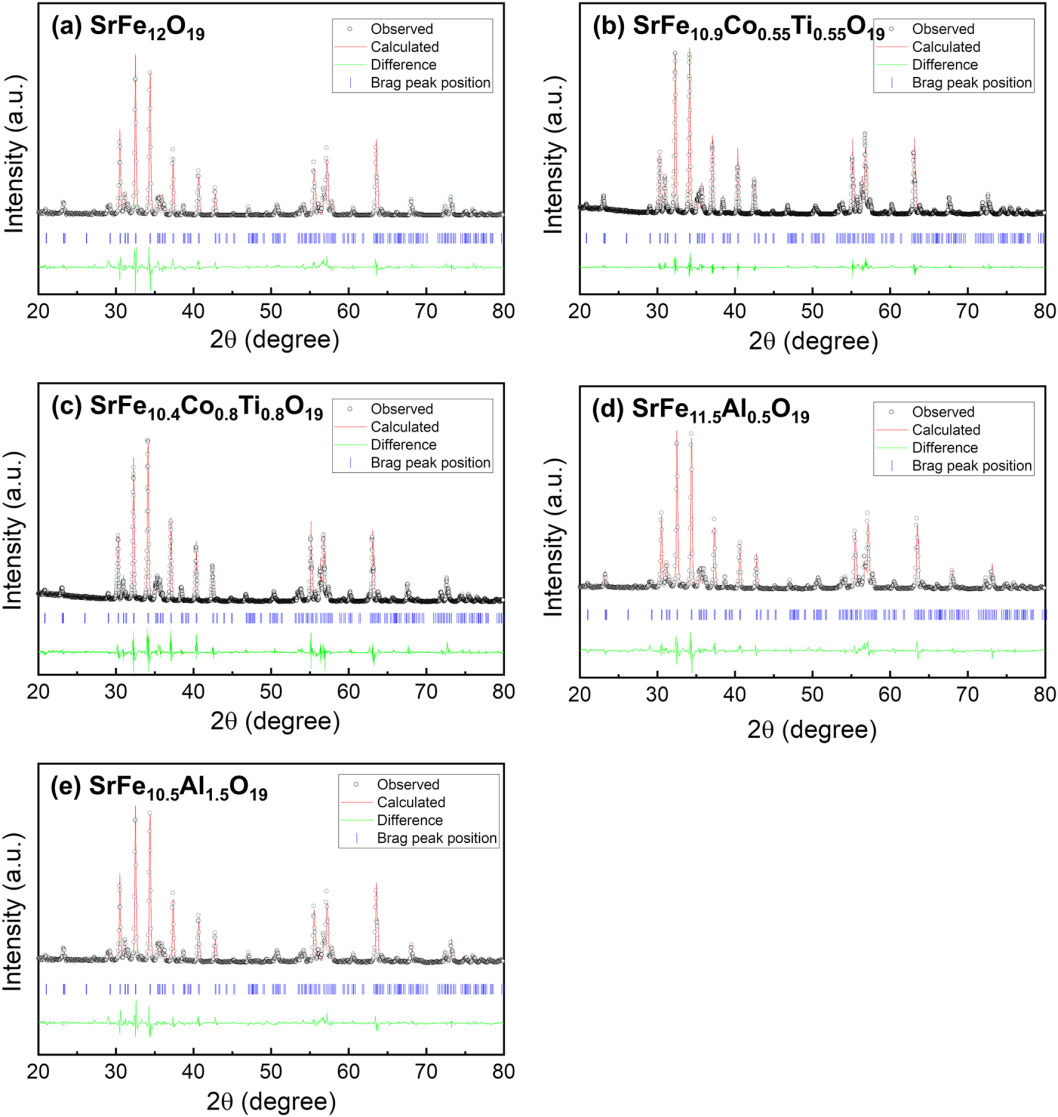
Refined X-ray diffraction patterns of **a** pure SrM, **b, c** Co–Ti doped SrM, and **d, e** Al doped SrM powder

Figure 2 displays the refined XRD patterns of pure (SrFe_12_O_19_) SrM, Co–Ti doped SrM, and Al doped SrM powder, respectively. The XRD data of all synthesized SrM samples were analyzed using the Rietveld refinement process with the Fullprof suite software package. As depicted in Fig. 2, all diffraction peaks are indexed to a single phase of the M-type hexagonal structure with P63/mmc space group. Furthermore, the experimental results for all samples agreed well with the corresponding simulated XRD pattern, as evidenced by the high goodness of fit parameters χ^2^, as shown in Table 1. These outcomes support the conclusion that the Fe sites of SrFe_12_O_19_ were successfully substituted by Co–Ti and Al atoms for all dopant concentrations without any secondary phase.

**Table 1:** Calculated lattice constants of doped SrMs and obtained fitting parameter value

Composition	Lattice constants			*R*-factors		
	a (Å)	c (Å)	V (Å)	R_p_ (%)	R_wp_ (%)	χ^2^
SrFe_12_O_19_	5.889	23.086	693.742	20.0	26.5	1.29
SrFe_10.9_Co_0.55_Ti_0.55_O_19_	5.889	23.086	693.826	20.8	20.3	0.946
SrFe_10.4_Co_0.8_Ti_0.8_O_19_	5.890	23.065	693.430	22.0	23.2	1.20
SrFe_11.5_Al_0.5_O_19_	5.864	22.931	683.481	18.5	18.2	0.917
SrFe_10.5_Al_1.5_O_19_	5.851	22.902	679.520	18.9	19.3	1.002

Table 1 also presents the lattice constants calculated from the refined XRD pattern. The lattice constant of Co–Ti doped SrMs remained unchanged with varying doping concentrations, whereas the lattice constant of Al doped SrMs decreased with increasing doping concentration. This phenomenon could be attributed mainly to the distinct ionic radii of Co^2+^, Ti^4+^, and Al^3+^. The ionic radii of Co^2+^ and Ti^4+^ are 0.745 and 0.605 Å, respectively, resulting in an average radius of 0.675 Å for co-substitution, which is comparable to the Fe^3+^ ion’s radius of 0.645 Å. On the other hand, the ionic radius of Al^3+^ is 0.535 Å, which is only 83% of the Fe^3+^ ionic radius, leading to a shrinkage of the crystal lattice.

**Fig. 3:**
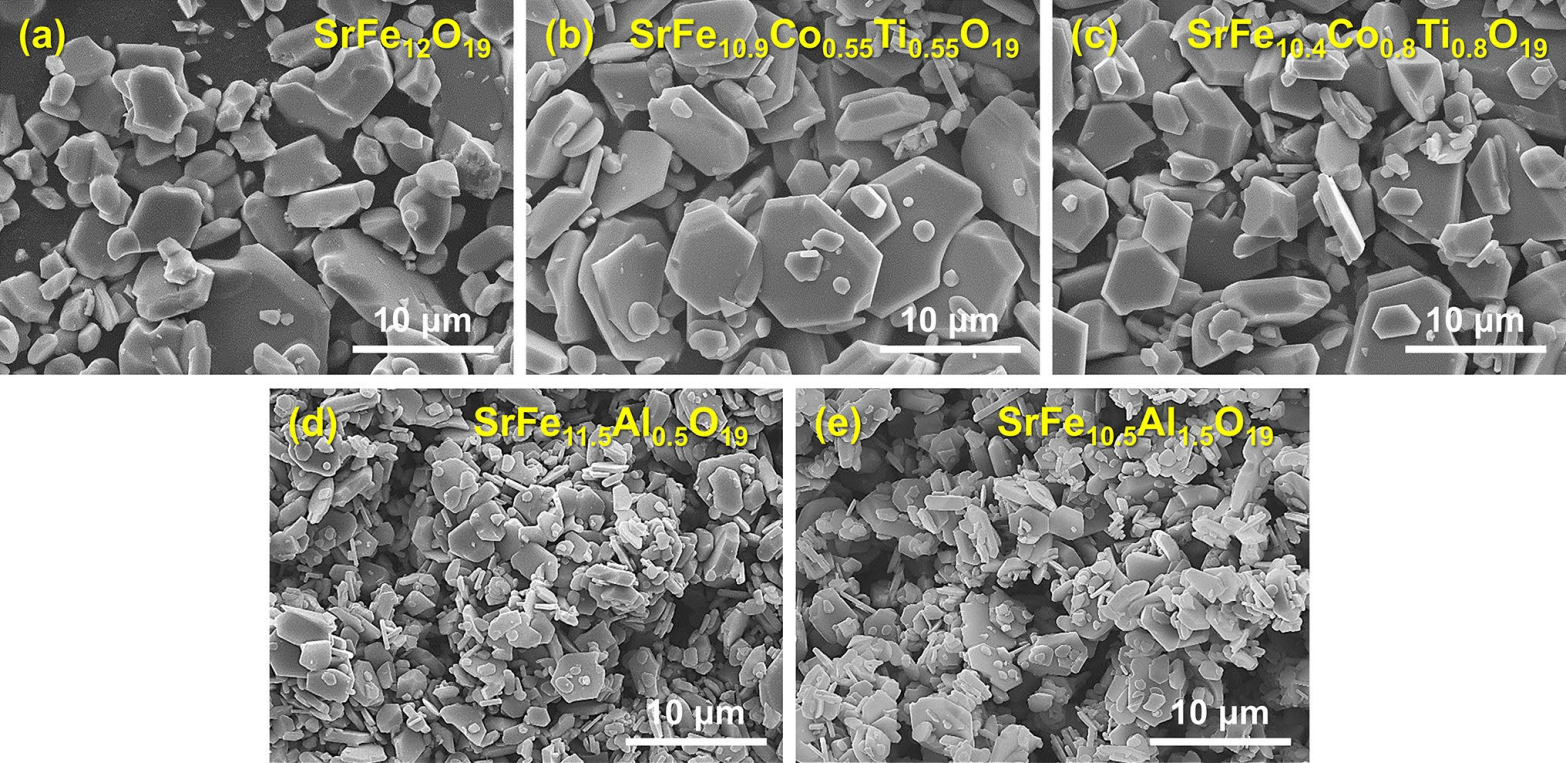
SEM images of **a** pure SrM, **b, c** Co-Ti doped SrM, and **d, e** Al doped SrM powders

Figure 3 illustrates the SEM images of the pure, Co-Ti doped, and Al doped SrM powders with different doping concentrations. All samples were calcined at 1250 °C for 3 h in molten NaCl flux for rapid mass diffusion, which facilitated crystal growth. Interestingly, Co–Ti doped SrM powders show a well-shaped hexagonal plate-like structure, and Al doped SrM powders show smaller particle sizes than pure SrM powders.

**Fig. 4:**
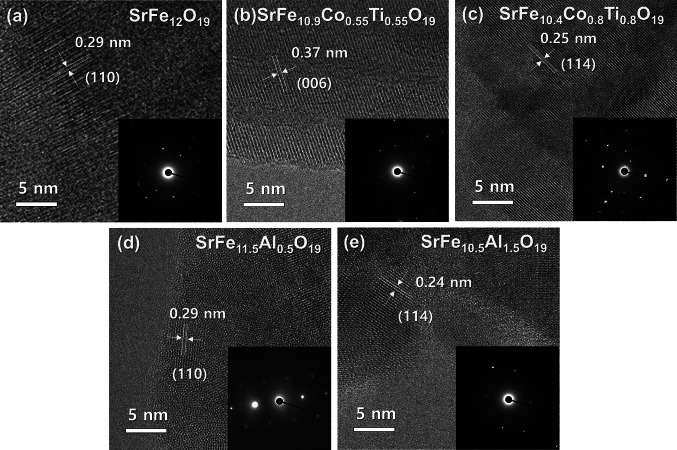
HR-TEM images and SEAD patterns(inset) of **a** pure SrM, **b, c** Co-Ti doped SrM, and **d, e** Al doped SrM powders

High-resolution TEM (HR-TEM) images and selective area electron diffraction (SEAD) patterns were also obtained to examine the detailed microstructure of Co-Ti doped and Al doped SrM powder. As shown in Fig. 4, all HR-TEM images shows distinct lattice fringes. The calculated lattice distances of 0.25, 0.29, and 0.37 nm are indexed to the (114), (110), and (006) planes of M-type crystal structure, respectively [63, 64]. The SEAD patterns (insets in Fig. 4) of all SrM exhibited hexagonal symmetry, which is consistent with the hexagonal crystal structure of the SrM phase. In addition, the simple dot SAED patterns of the pure and doped SrM imply their single crystalline nature. Overall, these results suggest that all doped SrM powders exist as a single hexaferrite phase, which supports the previous XRD results.

### Chemical State of Doped M-type Strontium Ferrites

**Fig. 5:**
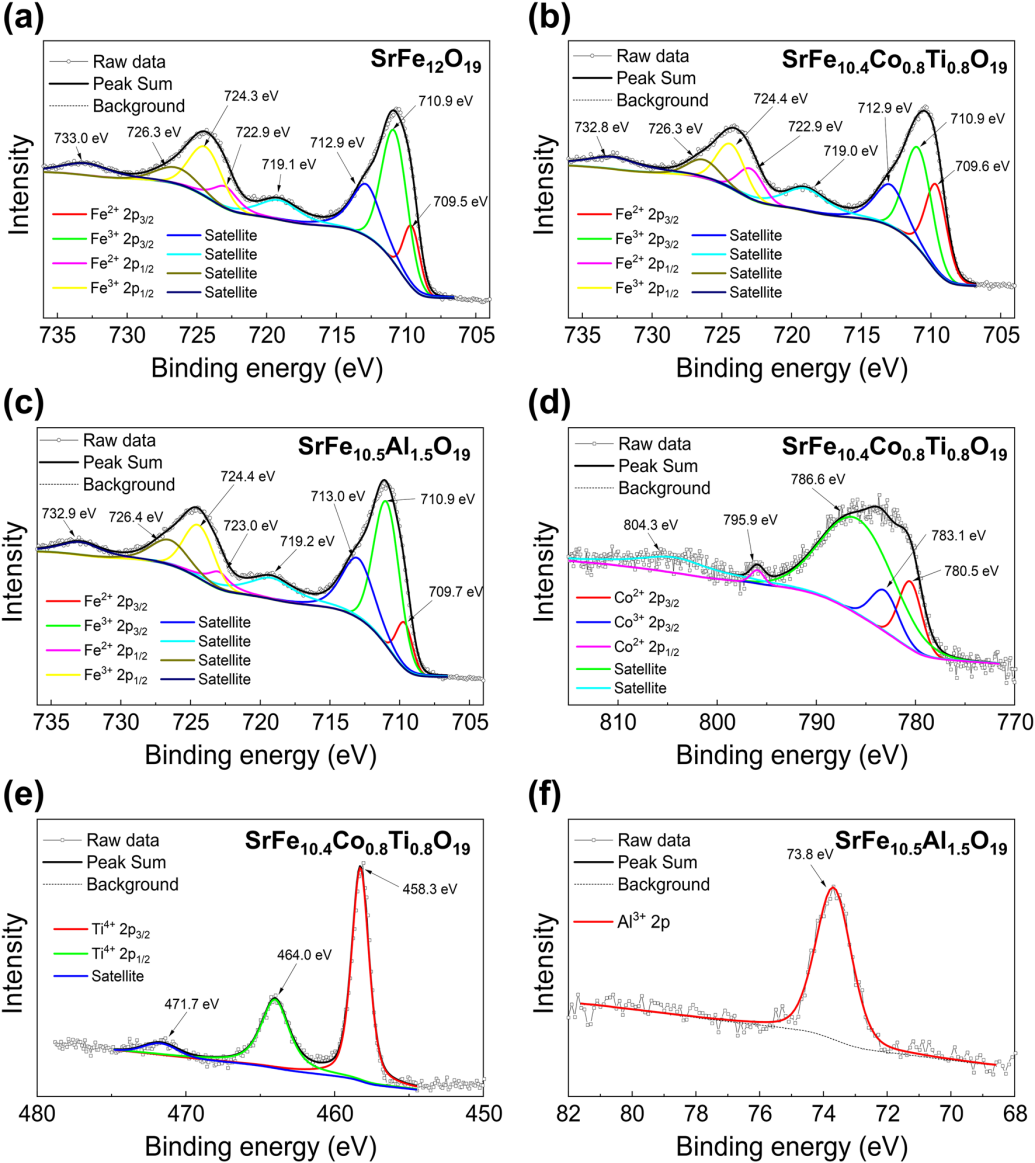
High resolution XPS spectra of pure and doped SrM. **a** pure Fe 2*p* spectra, **b** Co-Ti doped Fe 2*p* spectra, **c** Al doped Fe-2*p* spectra, **d** Co-Ti doped Co 2*p* spectra, **e** Co-Ti doped Ti 2*p* spectra and **f** Al doped Al 2*p* spectra

To investigate changes in the chemical bonding or valence state of SrM induced by Co–Ti or Al doping, XPS was employed to analyze the binding energy of SrM. The high-resolution XPS spectra of pure, Co–Ti doped, and Al doped SrM are presented in Fig. 5. The spectra were calibrated using the standard binding energy of C 2*s* (284.6 eV), and were fitted with a mixture of Lorenzian and Gaussian functions, along with a Shirley background function.

The high-resolution Fe 2*p* spectrum of pure, Co–Ti, and Al doped SrM is presented in Fig. 5a-c. The spectra exhibit two main peaks at 711 and 724 eV, corresponding to Fe 2*p*_3/2_ and Fe 2*p*_1/2_, respectively. These peaks can be further deconvoluted into three peaks each, corresponding to Fe^2+^, Fe^3+^, and a satellite peak. Notably, the peak area of the Fe^2+^ ion in Co–Ti doped SrM is larger than that in pure or Al doped SrM, which is believed to be due to the higher Fe^2+^ ion content of Co–Ti doped SrM as a result of charge balance compensation by doping of Ti^4+^ ions with higher valence than three. Previous studies have also reported similar results for M-type ferrites doped with higher valence ions such as Zr^4+^ or Nb^5+^ [53, 55].

The XPS spectra of Fig. 5d, f shows Co 2*p* and Ti 2*p* spectra of Co-Ti doped SrM, and Al 2*p* spectra of Al doped SrM, respectively. The deconvolved peaks in all spectra are consistent with previously reported data [60, 65, 66]. The presence of trivalent Co^3+^ ions in Co-Ti doped SrM is attributed to the charge transfer between Fe^3+^ and Co^2+^ ions (Fe^3+^  + Co^2+^  ↔ Fe^2+^  + Co^3+^). These XPS analysis results provide further evidence that Co–Ti and Al doped SrM have been successfully prepared.

### Magnetic Properties of Doped M-type Strontium Ferrites

**Fig. 6:**
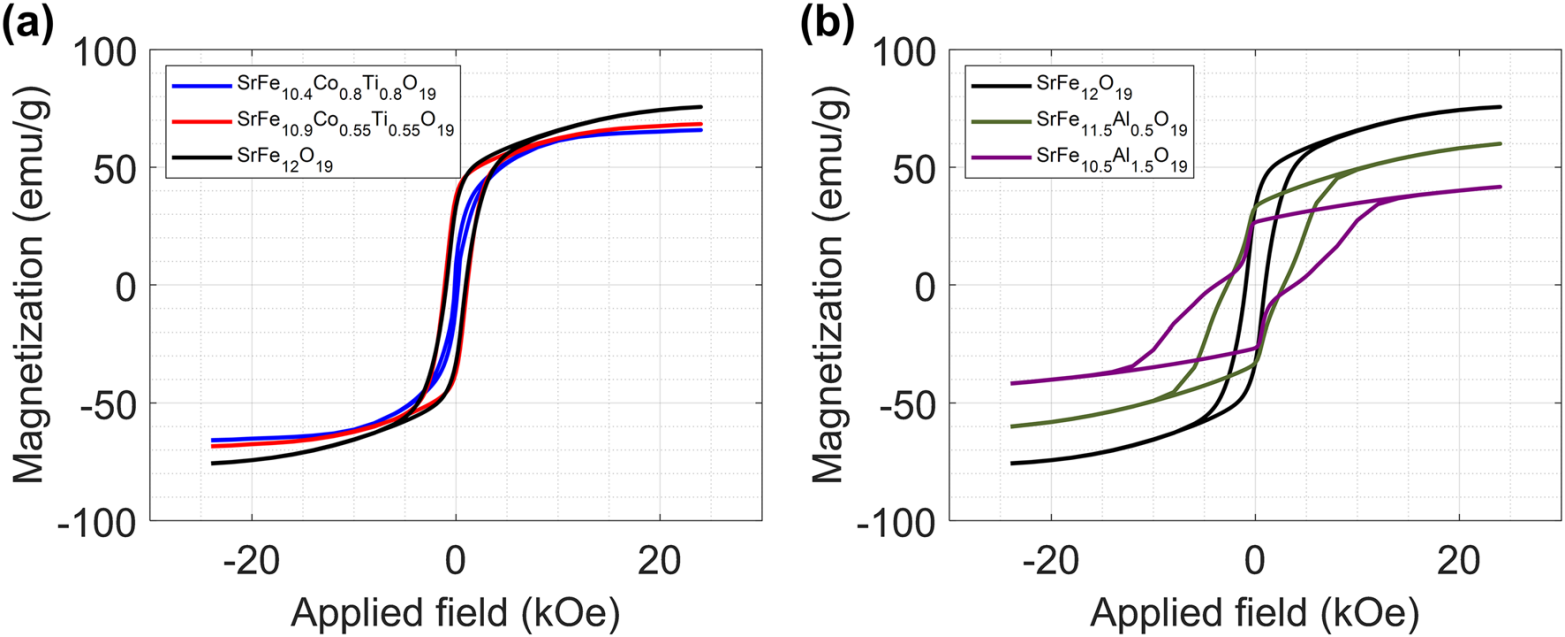
Magnetic hysteresis loop of pure, **a** Co-Ti doped, and **b** Al doped SrM powders

Figure 6a-b show the magnetic hysteresis loops of the Co-Ti doped and Al doped SrM powders at room temperature, respectively. Additionally, the detailed saturation magnetization (*M*_S_) and coercive field (*H*_C_) are presented in Table 2. Interestingly, pure and Co–Ti doped SrMs exhibited the typical hysteresis loop of single-phase magnetic materials, while the Al doped sample showed wasp-waisted hysteresis loops. This wasp waist hysteresis implies the presence of a small amount of secondary magnetic phase, but the existence of the secondary phase could not be confirmed by the XRD diffraction pattern in this study.

**Table 2:** Saturation magnetization and coercive field of doped SrM powders

Composition	*M*_S_ (emu/g)	*H*_C_ (Oe)
SrFe_12_O_19_	75.68	952
SrFe_10.9_Co_0.55_Ti_0.55_O_19_	68.74	1104
SrFe_10.4_Co_0.8_Ti_0.8_O_19_	65.86	161
SrFe_11.5_Al_0.5_O_19_	60.04	2757
SrFe_10.5_Al_1.5_O_19_	41.72	3843

The value of *M*_S_ for pure SrM is 75.68 emu g^−1^, which is similar to the theoretical value (~ 73 emu g^−1^) [67]. As shown in Fig. 6 and Table 2, the *M*_S_ of CoTi doped SrM powder decreases slightly with increasing dopant concentrations. However, for Al doped SrM powder, a noticeable decrease in the *M*_S_ is observed with increasing doping concentrations. This different behavior of the *M*_S_ for doped SrM powder is associated with different preferential site occupancies for Co–Ti and Al ions. For M-type ferrite, Fe^3+^ ions occupy 5 different lattice sites, which are named 2a, 2b, 12 k, 4*f*^1^ and 4*f*^2^ (Fig. S1). 2a, 12 k and 4*f*^2^ are octahedral sites, 4*f*^1^ is a tetrahedral site and 2b is a trigonal bipyramidal site. The 2a, 2b and 12 k sites are spin-up, while 4*f*^1^ and 4*f*^2^ are spin down; thus, the total magnetization of M-type ferrite is contributed by the excess spin-up magnetic moment [67]. The magnetic moments of Fe^3+^, Co^2+^, Ti^4+^ and Al^3+^ are 5 μ_B_, 3 μ_B_, 0 μ_B_, and 0 μ_B_, respectively. Because Co^2+^, Ti^4+^ and Al^3+^ ions show a lower magnetic moment than Fe^3+^ ions or zero magnetic moment, we can expect that the *M*_S_ will decrease if the dopant ions occupy spin-up sites such as 2a, 2b or 12 k and increase if the dopant ions occupy spin-down sites such as 4*f*^1^ and 4*f*^2^.

In previously reported Mössbauer spectra analysis, Co^2+^ and Ti^4+^ ions in co-doped M-type ferrites prefer to occupy 4*f*^2^ (spin down) and 2b (spin up), respectively [68–71]. Therefore, these opposite spin directions will cause a reduced dilution effect of the Co–Ti substitution for total magnetization, and finally, the *M*_S_ of Co–Ti doped SrM slightly decreases with higher doping concentrations. However, according to the Mössbauer spectra analysis and first-principles total-energy calculations, Al^3+^ ions for M-type ferrites prefer to occupy 12 k (spin up) and 2a (spin up), respectively [72–74]. Therefore, the decrease in the *M*_S_ for Al doped SrM can be explained by substitution of non-magnetic ions for magnetic Fe^3+^ ions at the up-spin site, which reduced the total magnetization.

The coercive field (*H*_C_) of doped SrM also showed different changing behaviors depending on the doping element and doping concentration. The *H*_*C*_ decreased as the doping concentration increased in Co–Ti doped SrM, resulting in soft magnetic properties; however, the *H*_C_ increased as the doping concentration increased in Al doped SrM, resulting in hard magnetic properties. This change in the coercive field can be explained by a difference in the particle size and a change in the magnetic anisotropy. The coercive field of a magnetic particle is generally related to the critical single domain size (*D*_C_). According to previous studies, the critical single domain size of M-type ferrite is approximately 1 μm, and as the ferrite particle size increases or decreases, the coercive field decreases [59, 75]. As shown in Fig. 3, Co–Ti doped SrM powder mostly consists of hexagonal plate-shaped particles of 5 μm or larger, but Al doped SrM powder contains a large amount of particles with sizes of 1 μm. Therefore, it is considered that the *H*_C_ of Al doped SrM, whose particle size is similar to the critical single domain size, is greater than that of Co–Ti doped SrM.

The *H*_C_ of doped SrM is also changed by the magnetic anisotropy change due to the doping element and concentration. M-type ferrite has a large c-axis anisotropy, and the magnitude of this anisotropy varies depending on the substitution site of the doped element. The decreased *H*_C_ of Co–Ti doped SrM is related to the spin rearrangement by Co^2+^ and Ti^4+^ ion substitutions. In particular, Co^2+^–Ti^4+^ substituted at the 2b site reduces the uniaxial anisotropy so that the coercive field is greatly reduced according to the Co–Ti doping concentration [65, 76, 77]. The increased *H*_C_ of Al doped SrM is related to an enhancement of the magneto-crystalline anisotropy constant due to the substituted Al^3+^ ions at the 12 k site [58, 59, 78].

### Electromagnetic Properties of the Doped M-type Strontium Ferrites

The incorporation of magnetic materials in EMI shielding materials provides various EMI absorption mechanisms including (1) hysteresis, (2) domain wall resonance, (3) eddy current, and (4) natural ferromagnetic resonance (FMR) [79]. In an oscillating magnetic field, the material magnetization is reversed, resulting in the dissipation of electromagnetic energy as heat. This dissipated energy is proportional to the area of the hysteresis loop. The oscillating magnetic field also induces a pressure that causes the domain walls in the magnetic material to oscillate. The oscillating domain wall dissipates its energy through the interaction with magnetic domains and the magnetic particle grain boundary [80]. Eddy current induced around a magnetic particle is dissipated as heat through Joule effect, as it is a current flowing through a conductor. However, these three mechanisms are known to be effective in low frequency range under several GHz [81]. For microwave and mmWave applications, FMR is the most major contributor [82]. The resonance absorbs EMI and converts it into local heating of the material, resulting high magnetic loss tangent and imaginary permeability at the FMR frequency.

**Fig. 7:**
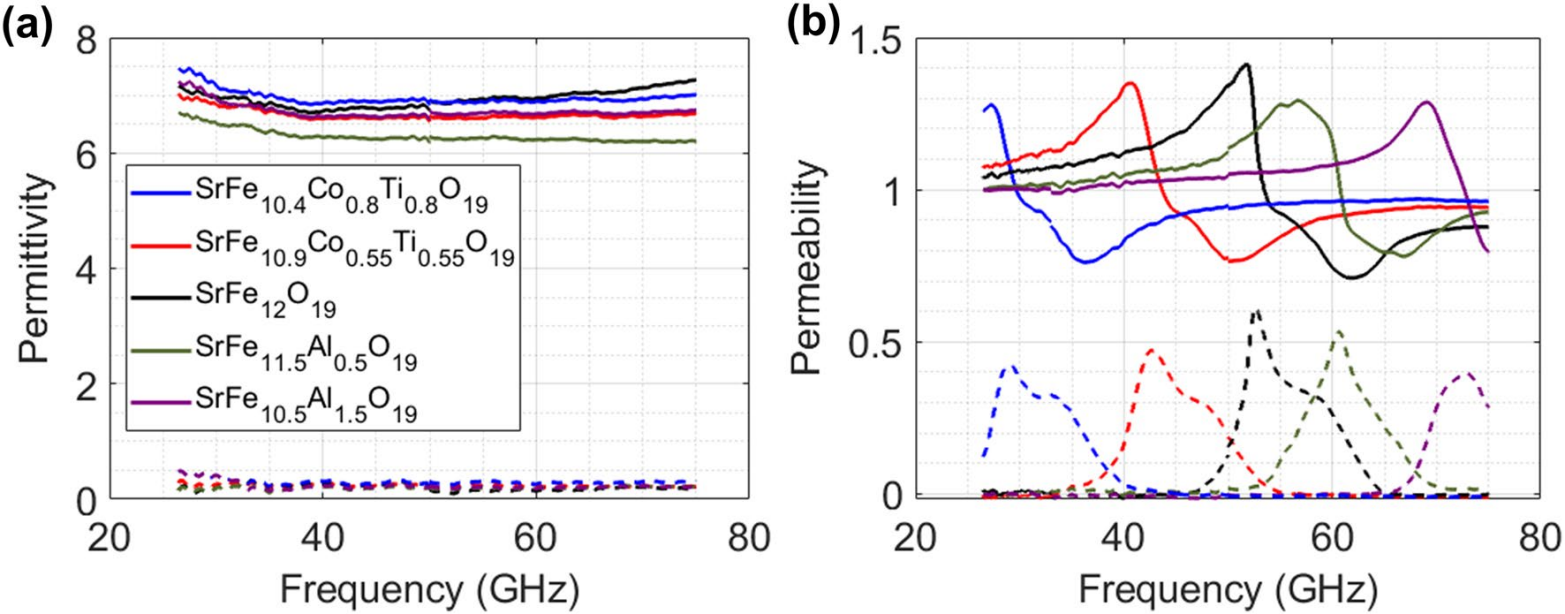
**a** Permittivity and **b** permeability of pure, Co-Ti doped, and Al doped SrM composites

Figure 7 shows the complex permittivity (*ε*) and permeability (*μ*) of pure, Co–Ti doped and Al doped SrM composites. These composites consist of 70 wt% SrM and 30 wt% thermoplastic polyurethane (TPU). In each figure, the real parts and the imaginary parts are presented as solid lines and dashed lines, respectively. The real (*ε′*) and imaginary (*ε′′*) parts of the permittivity of all SrMs are not significantly different. As shown in Fig. 7b, all synthesized samples show clear FMR peaks of real (*μ′*) and imaginary (*μ′′*) permeability, but the FMR frequencies are different with the doping element and concentration. From Fig. 7b, it can be seen that the doping of Co–Ti shifts the FMR frequency to a lower frequency band, whereas Al doping pushes the FMR frequency to a higher frequency band. Furthermore, it was confirmed that the degree of the frequency shift increased with doping concentrations.

It is known that the FMR frequencies of the M-type ferrites are closely related to the magnetic anisotropy. The FMR frequency (*f*_r_) is proportional to the anisotropy field (*H*_a_) as:

9 $${f}_{\mathrm{r}}= \frac{\gamma }{2\pi }{H}_{\mathrm{a}}=1.4g{H}_{\mathrm{a}}$$

where *γ* is the gyromagnetic ratio, and *g* is the Lande factor. Therefore, it can be expected that the substitution of Co-Ti decreases *H*_a_, whereas the substitution of Al increases *H*_a_. In fact, the contribution to the magnetic anisotropy of M-type hexaferrite differs at each Fe^3+^ site in the crystal lattice. According to previous reports, the replacement of Fe^3+^ ions at the 2b and 4f2 sites has the greatest contribution to the anisotropy field [83, 84]. The anisotropy field can be expressed by *K*_1_ and *M*_S_ as:

10 $${H}_{\mathrm{a}}= \frac{2{K}_{1}}{{M}_{\mathrm{S}}}$$

where *K*_1_ is magnetocrystalline anistropy constant. The calculated *K*_1_ for a single Fe^3+^ ion and the degree of contribution to *H*_a_ at each site are 1.4, 0.51, 0.23, 0.18, and − 0.18 for the 2b, 4*f*^2^, 2a, 4*f*^1^, and 12 k sites, respectively [85]. In other words, when compared to other sites, the Fe^3+^ ions at the 2b site contribute the most to *H*_a_, while the Fe^3+^ ions at the 4*f*^1^ and 12 k site contribute the least *H*_a_.

As discussed above, the Co^2+^ and Ti^4+^ ions prefer to occupy 2b and 4*f*^2^, respectively. When Co–Ti is doped, Co^2+^ and Ti^4+^ ions replace Fe^3+^ ions in the 2b and 4*f*^2^ positions, which have high *K*_1_ values. As a result, the *K*_1_ value decreases significantly, but the *M*s value decreases relatively less due to the opposite spin directions of the substituted site. Therefore, the *H*_a_ value of the Co–Ti doped SrM decreases, and the FMR frequency shifts to the low frequency band.

Unlike Co–Ti ions, Al^3+^ ions prefer to occupy 12 k and 2a sites. In particular, at high calcination temperatures, Al^3+^ ions are much more likely to occupy the 12 k site than the 2a site [74]. Since the *K*_1_ value of a single Fe^3+^ at 12 k sites is negative, the total *K*_1_ value of Al doped M-type ferrite will be increased. However, *M*_S_ is significantly decreased when nonmagnetic Al^3+^ ions are substituted for the up-spin 12 k and 2a sites. Therefore, the *H*_*a*_ value of Al doped SrM increases, and the FMR frequency shifts to a high frequency band.

Another factor affecting *K*_1_ is the lattice parameters. It is known that the *K*_1_ value of M-type ferrite is affected by the bond length between iron at the 2b (Fe_2_) site and adjacent O atoms (O_3_) [58]. As previously discussed, the lattice parameters of Al doped SrM were reduced due to the smaller ionic radius of Al^3+^ ions relative to Fe^3+^ ions. The crystal lattice contraction by Al^3+^ substitution decreases the bond length between Fe_2_–O_3_, which induces enlargement of the superexchange interaction. This increased superexchange interaction enhances the magnetic anisotropy, shifting the FMR frequencies to higher frequencies.

## EMI Shielding Films with Multi-band Ultralow Reflection

### EMI Shielding Film Design

**Fig. 8:**
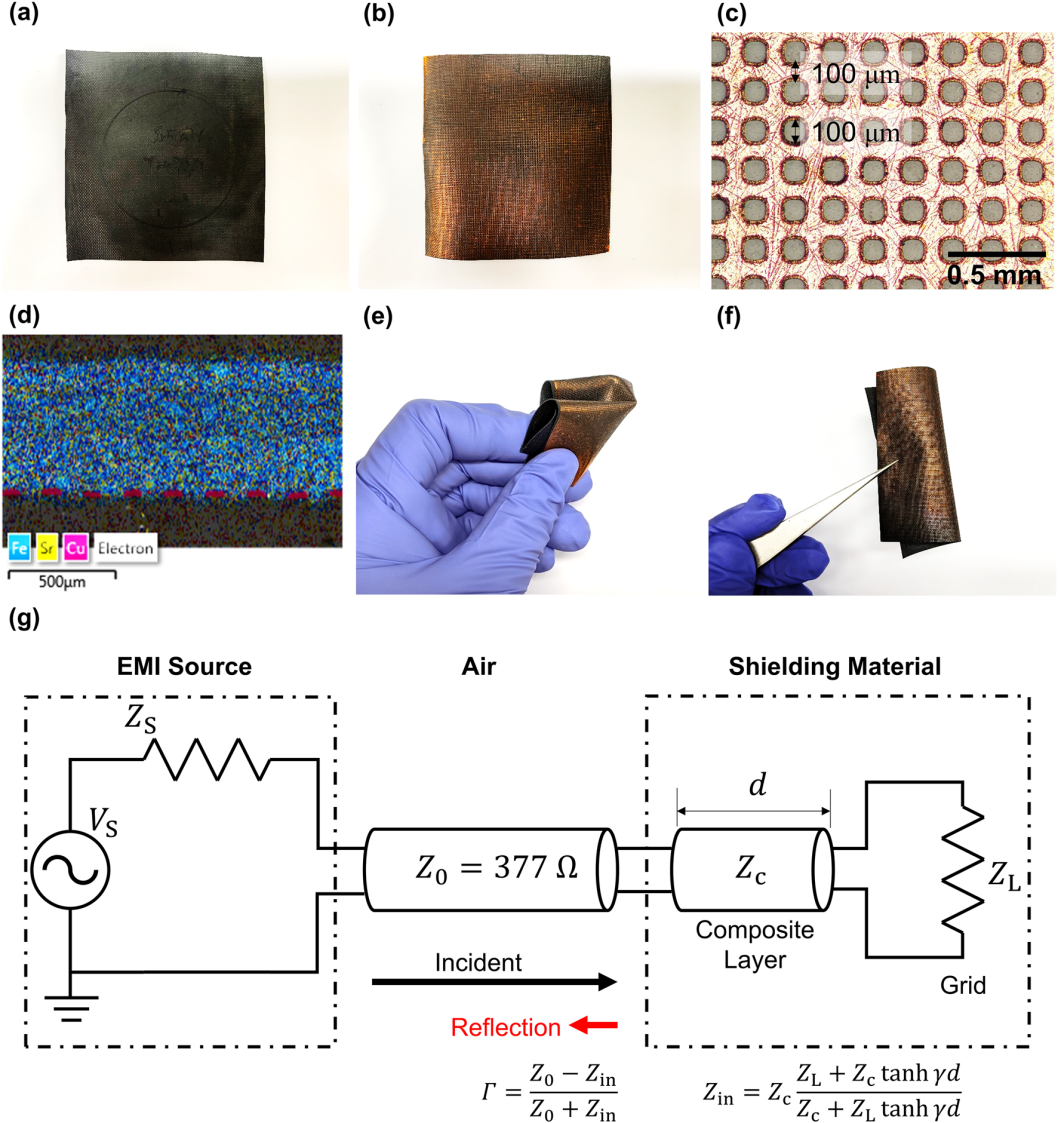
The proposed EMI shielding film design: **a, b** Front and back view of the film. **c** Optical microscopic image of the Cu grid. **d** SEM–EDS cross section image of the film. **e, f** Folded and rolled shielding films. **g** Transmission line model of the proposed shielding film

Based on SrM, the EMI shielding film is proposed with a magnetic composite layer and a conductive grid (Fig. 8a, b). The composite layer is prepared by dispersing SrM powders to the TPU. Then, a 10 μm thick Cu grid is attached underneath the composite layer via hot pressing. This Cu grid is prepared through eletroforming to achieve a fine conductive grid with a 100 μm grid width and 100 μm gap between each grid (Fig. 8c). The cross-sectional SEM‒EDS image in Fig. 8d shows that the composite layer with SrM (Yellow: Sr, Blue: Fe) and the Cu grid (Magenta) are bonded well to each other. According to ASTM D3359 Method B cross-cut adhesion test, the Cu grid has 4B/5B adhesion with the composite layer, which meets the standards of practical industrial applications (Fig. S2) [86]. Since the shielding film is sub-millimeter thin and TPU-based, the film is quite flexible and still intact even after folding and rolling (Fig. 8e, f).

The EMI shielding performance of a shielding material is composed of two parts: reflection and absorption. The EMI reflection from the shielding film can be explained with a transmission line model of electromagnetic waves (Fig. 8g). When the incident EMI faces the shielding film surface, the amount of reflected EMI, or reflection coefficient *Г*, is defined as [87]:

11 $$\Gamma =\frac{{Z}_{0}-{Z}_{\mathrm{in}}}{{Z}_{0}+{Z}_{\mathrm{in}}}$$

where ${Z}_{0}$ is the electromagnetic impedance of air, and ${Z}_{\mathrm{in}}$ is the input impedance of the shielding film system. Therefore, it is important to minimize the impedance difference between the air and the shielding film to minimize the reflected amount of the EMI. As the shielding film consists of a composite layer and a grid, ${Z}_{\mathrm{in}}$ is given as [29, 88]:

12 $${Z}_{\mathrm{in}}={Z}_{\mathrm{c}}\frac{{Z}_{\mathrm{L}}+{Z}_{\mathrm{c}}\mathrm{tanh}\gamma d}{{Z}_{\mathrm{c}}+{Z}_{\mathrm{L}}\mathrm{tanh}\gamma d}$$

13 $${Z}_{\mathrm{c}}={Z}_{0}\sqrt{\frac{\mu }{\varepsilon }}$$

14 $$\gamma =\mathrm{j}2\uppi f\frac{\sqrt{\mu \varepsilon }}{c}$$

where ${Z}_{\mathrm{c}}$ is the electromagnetic impedance of the composite layer; ${Z}_{\mathrm{L}}$ is the electromagnetic impedance of the conductive grid; $d$ is the thickness of the composite layer; $\mu$ and $\varepsilon$ are the complex permeability and permittivity of the composite layer, respectively; $f$ is the EMI frequency; *j* is the imaginary unit; *γ* is the electromagnetic wave propagation constant and $c$ is the speed of light in free space.

From the above equations, given $\mu$, $\varepsilon$, $d$, and ${Z}_{\mathrm{L}}$, a single local minimum of *Г* exists at a specific frequency if $\mu$ and $\varepsilon$ are frequency independent. However, if there is a sudden increase in $\mu$ and $\varepsilon$, as proposed with FMR frequency tunable SrMs (Fig. 7), an additional impedance matching occurs at the FMR frequency, and additional local minimum appears close to the FMR frequency (Fig. S3). This enables ultralow reflection at multiple frequencies and low reflection in a wider frequency band. It should be noted that the *Г* depends on not only permeability of the composite layer but also its permittivity. To adjust the real and imaginary permittivity for optimized *Г*, dielectric filler materials, e.g., silver nanowire [89], graphene [90], or carbon black [91], can be included to the composite layer. Carbon nanotubes are one of the most favorable dielectric filler thanks to their lightweight nature and high permittivity change with a small amount of their incluson.

Therefore, by carefully tailoring the composite layer characteristics and grid characteristics, including the FMR frequency of SrMs in the composite layer, it is possible to have ultralow reflection at the desired two frequency bands: one corresponding to the FMR frequency and the other corresponding to the film composition. Additionally, the grid impedance can be controlled by changing the grid width and gap. As the conductive area increases, with a wider grid and narrower gap between them, the grid becomes similar to a conductive metal layer, and its impedance decreases to zero. On the other hand, with a narrower grid and wider gap, a smaller portion of the conductive material increases the ${Z}_{\mathrm{L}}$. It has been investigated that a proper selection of ${Z}_{\mathrm{L}}$ helps decrease the reflection of a grid-based shielding film [29]. Then, the SER is given as:

15 $$\mathrm{SER}=-10\mathrm{log}\left(1-{\left|{S}_{11}\right|}^{2}\right)=-10 \mathrm{log}(1-{\left|\Gamma \right|}^{2})$$

where S_11_ is a scattering parameter corresponding to EMI reflection. For more details regarding Eq. (15), the experimental section can be referenced.

The other part of EMI shielding, absorption, can be explained as electromagnetic wave attenuation in a lossy material. Due to the dielectric and magnetic loss of the material, the electromagnetic wave amplitude decays exponentially as it passes through the material [92]. Then, the absorption shielding effectctiveness (SEA) of the material is defined as [4]:

16 $$\mathrm{SEA}=-10{\mathrm{log}}_{10}\left(\frac{{\left|{S}_{21}\right|}^{2}}{1-{\left|{S}_{11}\right|}^{2}}\right)=20{\mathrm{log}}_{10}\left|{e}^{\gamma t}\right|=20\alpha t{\mathrm{log}}_{10}e$$

where $\alpha$ is the attenuation constant of the electromagnetic waves in the material, and *t* is the material thickness. For a non-conducting material, e.g., the composite layer of the proposed shielding film, the attenuation constant is given as [38]:

17 $$\alpha {\kern 1pt} = {\kern 1pt} {\text{Re}}\left( \gamma \right){\kern 1pt} = {\kern 1pt} \frac{\pi f}{c}\sqrt {2\mu^{\prime } \varepsilon^{\prime } } \sqrt {\frac{{\mu^{\prime \prime } \varepsilon^{\prime \prime } }}{{\mu^{\prime } \varepsilon^{\prime } }}{\kern 1pt} - {\kern 1pt} 1{\kern 1pt} + {\kern 1pt} \sqrt {\left( {\frac{{\mu^{\prime \prime } }}{{\mu^{\prime } }}} \right)^{2} + \left( {\frac{{\varepsilon^{\prime \prime } }}{{\varepsilon^{\prime } }}} \right)^{2} + \left( {\frac{{\mu^{\prime \prime } \varepsilon^{\prime \prime } }}{{\mu^{\prime } \varepsilon^{\prime } }}} \right)^{2} {\kern 1pt} + {\kern 1pt} 1} }$$

From Eqs. (16) and (17), it is shown that the SEA increases with (1) thicker material or (2) higher dielectric/magnetic loss. The major energy transformation mechanism of dielectric lossy fillers, CNT in the proposed shielidng film, is Joule effect [93]. A given external electric field, the electromagnetic waves, displaces charges in the dielectric filler and causes dielectric polarization. This enables the dielectric filler to store electric potential energy. As this system can be considered as a circuit with a capacitor (the real part of permittivity) and a resistance (the imaginary part of permittivity), the stored electromagnetic energy is transformed and dissipated as heat at the resistance. On the other hand, the magnetic lossy fillers absorb electromagnetic waves by (1) hysteresis, (2) domain wall resonance, (3) eddy current, and (4) natural ferromagnetic resonance (FMR), and FMR is the most major contributor in mmWave applications. Therefore, it is important to increase the electromagnetic loss, especially with FMR in this study, to have a high SEA with less thickness. The synergetic effects between magnetic loss from ferromagnetic resonance of SrMs and dielectric loss from CNT as well as interfacial polarization in the composite layer are favorable for enhancement of EMI absorption capabilities [42]. These equations also show why foam materials need to be thicker, as they are electromagnetically less lossy with a high void content.

For a multilayer structure such as the proposed shielding film, the total SEA is known to be the sum of the SEAs of each layer. For a conducting material, e.g., the conductive grid of the proposed shielding film, the attenuation constant is

18 $$\alpha =\sqrt{\pi f\mu \sigma }$$

where *σ* is the electric conductivity. As the conductive grid shows more than 30 dB SEA thanks to its high conductivity, the total SE of the proposed shielidng film becomes higher with the conductive grid and high magnetic loss of the composite layer.

### 5G Telecommunication Application: 39/52 GHz Shielding Film

**Fig. 9:**
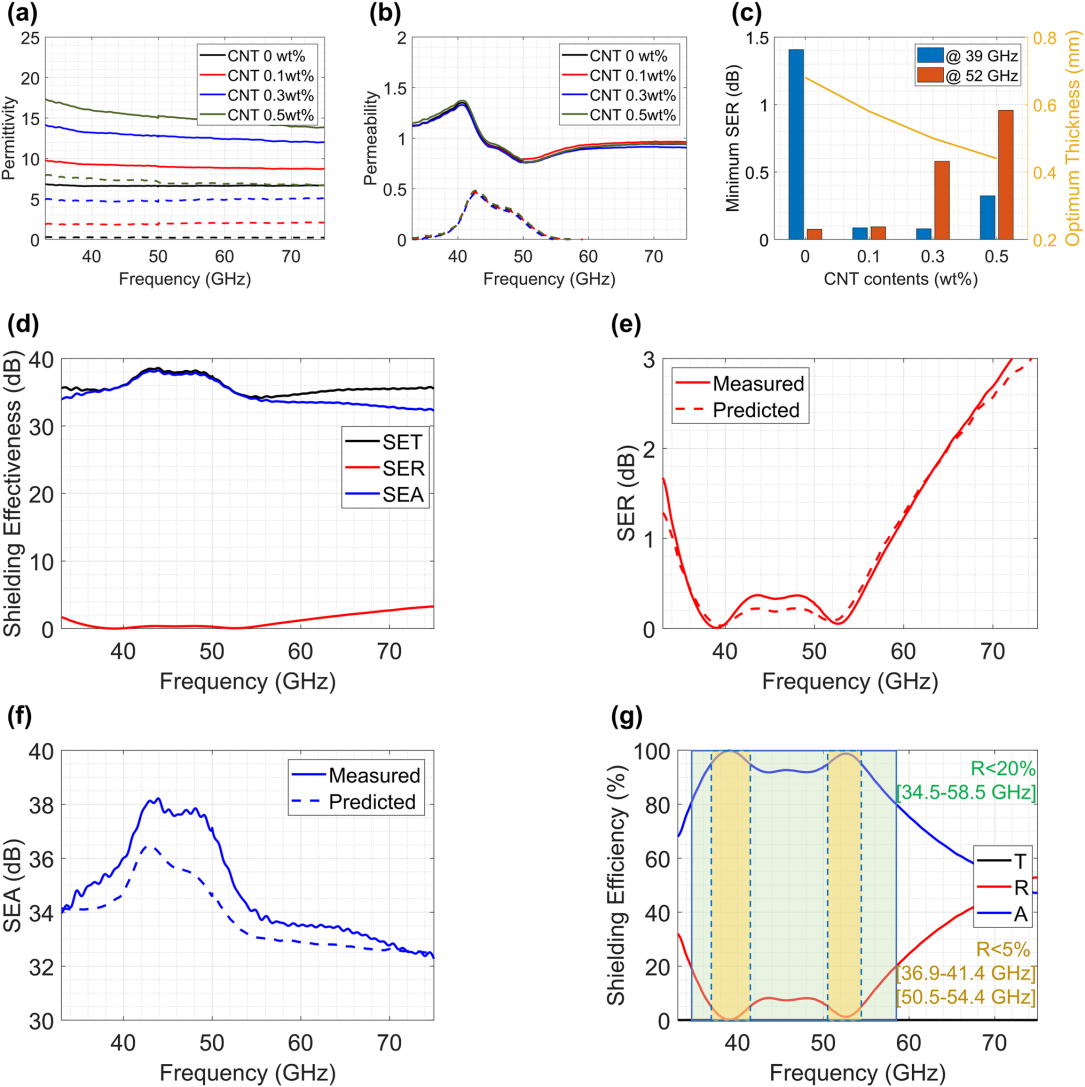
EMI shielding film for 5G telecommunication applications: **a** Complex permittivity and **b** permeability of composite layers with different CNT contents. **c** Estimated minimum SER of each specimen at 39 and 50 GHz. **d** Measured shielding effectiveness of the prepared EMI shielding film. Comparison of the measured and predicted **e** SER and **f** SEA. **g** The measured shielding efficiency of the prepared EMI shielding film

Based on the aforementioned design factors, two types of mmWave EMI shielding films with ultralow reflection are designed. The first film focuses on shielding 39 and 52 GHz frequency bands, which corresponds to future 5G wireless communication band candidates [94]. In particular, the 39 GHz band (37–40 GHz) is known as the 5G Band n260, and most major carriers in the US, including AT&T and Verizon, already acquired this band through a frequency band auction by the Federal Communication Commission [95]. The composite layer of this film includes 70 wt% SrFe_10.9_Co_0.55_Ti_0.55_O_19_ which is SrM with an FMR frequency near 39 GHz. The layer contains CNTs as well to control its permittivity to optimize the input impedance of Eq. (12). Figure 9a–b presents the measured complex permittivity and permeability of the composite layer with different amounts of CNTs. Both the real (solid lines in Fig. 9a) and imaginary (dashed lines in Fig. 9a) permittivity increase with additional CNT content, while the permeability does not change as CNTs are not magnetic materials. The SER of shielding films with each composite layer can be estimated using the proposed transmission line model and Eq. (15). Figure 9c presents the calculated minimum SER at 39 and 52 GHz with different CNT contents at the optimum layer thickness. It has been shown that the shielding film may have the lowest SER with 0.1 wt% CNTs and a 0.58 mm layer thickness.

Based on this estimation, the actual shielding film is fabricated, and its EMI shielding performance is evaluated. Figure 9d presents the measured SE at 33–75 GHz, and Fig. 9e and f provide closer looks of the SER and SEA, respectively. This film shows an exceptionally low SER of less than 0.1 dB at 39 and 52 GHz, while shielding more than 30 dB over the entire frequency range. The first minimum SER frequency (0.009 dB at 38.9 GHz) is located around the FMR frequency of the doped SrM, and the other minimum SER frequency (0.055 dB at 52.5 GHz) is determined with the layer composition. The SEA shows a high peak in the 40–50 GHz band due to the high magnetic loss around the FMR frequency. It should also be noted that the predicted SER and SEA using the theoretical model (Eqs. 15, 16), presented as dashed lines in Fig. 9e–f, match very well with the measured values (solid lines).

The outstanding shielding performance of the film is better presented with its high shielding efficiency (Fig. 9g). The film reflects only 0.2% and absorbs 99.7% of EMI at 39 GHz and reflects only 1.6% and absorbs 98.3% at 52 GHz. Moreover, not only at these specific frequencies, the film shows ultralow reflection of less than 5% in two bands, including each frequency (36.9–41.4 GHz and 50.5–54.4 GHz), and low reflection less than 20% in a broad frequency band (34.5–58.5 GHz). It should be noted that the first ultralow reflection band covers the whole n260 band (37–40 GHz). This implies that the proposed shielding film can solve EMI problems in 39 and 52 GHz telecommunication bands.

### Automotive Radar Application: 60/77 GHz Shielding Film

**Fig. 10:**
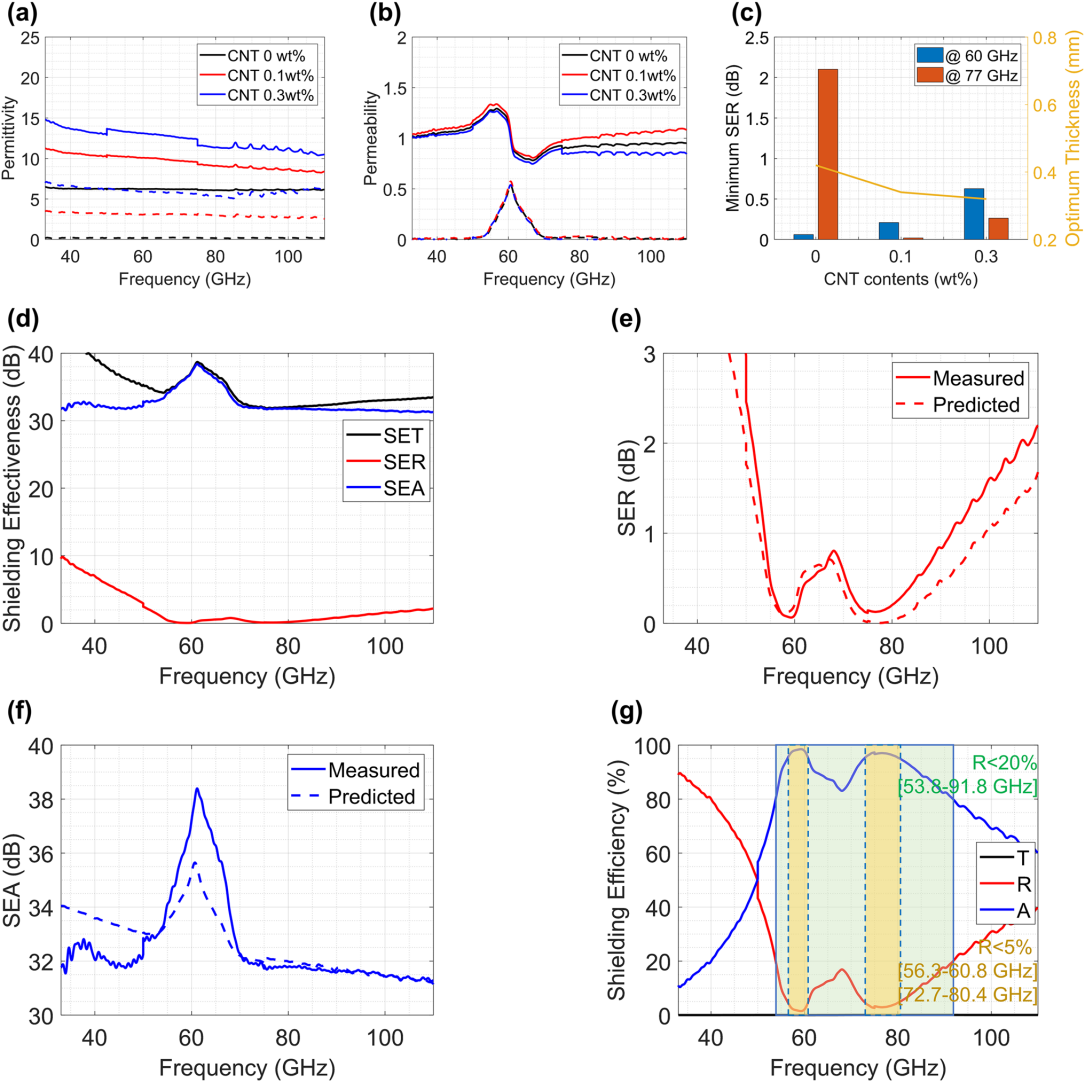
EMI shielding film for automotive radar applications: **a** Complex permittivity and **b** permeability of composite layers with different CNT contents. **c** Estimated minimum SER of each specimen at 60 and 77 GHz. **d** Measured shielding effectiveness of the prepared EMI shielding film. Comparison of the measured and predicted **e** SER and **f** SEA. **g** The measured shielding efficiency of the prepared EMI shielding film

The other film focuses on shielding 60 and 77 GHz frequency bands, which correspond to automotive radar bands. There is an increasing usage of radars for automobiles, and 77 GHz long range radar is essential for recently manufactured autonomous vehicles with ADAS [96]. 60 GHz radar is also of interest for multi-functional in-cabin monitoring systems, e.g., child presence detection, gesture detection, and driver vital monitoring [97]. The composite layer of this film contains 70 wt% SrFe_11.5_Al_0.5_O_19_ and SrM with an FMR frequency near 60 GHz. The layer also contains CNTs to control its permittivity. Figure 10a and b present the measured complex permittivity and permeability of the composite layer with different amounts of CNTs, respectively. According to the proposed transmission line model, the shielding film may have the lowest SER with 0.1 wt% CNTs and a 0.34 mm layer thickness (Fig. 10c).

Figure 10d presents the measured SE of the fabricated shielding film on 33–110 GHz with closer looks of the SER (Fig. 10e) and SEA (Fig. 10f). As in the previous example, the first minimum SER frequency (0.065 dB at 59.3 GHz) is located around the FMR frequency, and the other minimum SER frequency (0.12 dB at 74.8 GHz) is determined with the layer composition. The predicted SER and SEA also match very well with the measured values.

The efficiency is also presented in Fig. 10g. The film reflects only 1.5 and absorbs 98.4% of EMI at 60 GHz; moreover, it reflects only 2.9% and absorbs 97.0% at 77 GHz. In addition to these specific frequencies, the film shows ultralow reflection of less than 5% in two bands (56.3–60.8 GHz and 72.7–80.4 GHz) and low reflection less than 20% in a broad frequency band (53.8–91.8 GHz). This shows that the proposed shielding film can work as an effective EMI shielding film for a wide mmWave frequency range including 60 and 77 GHz automotive radar bands.

### Comparison with Previous Studies

**Fig. 11:**
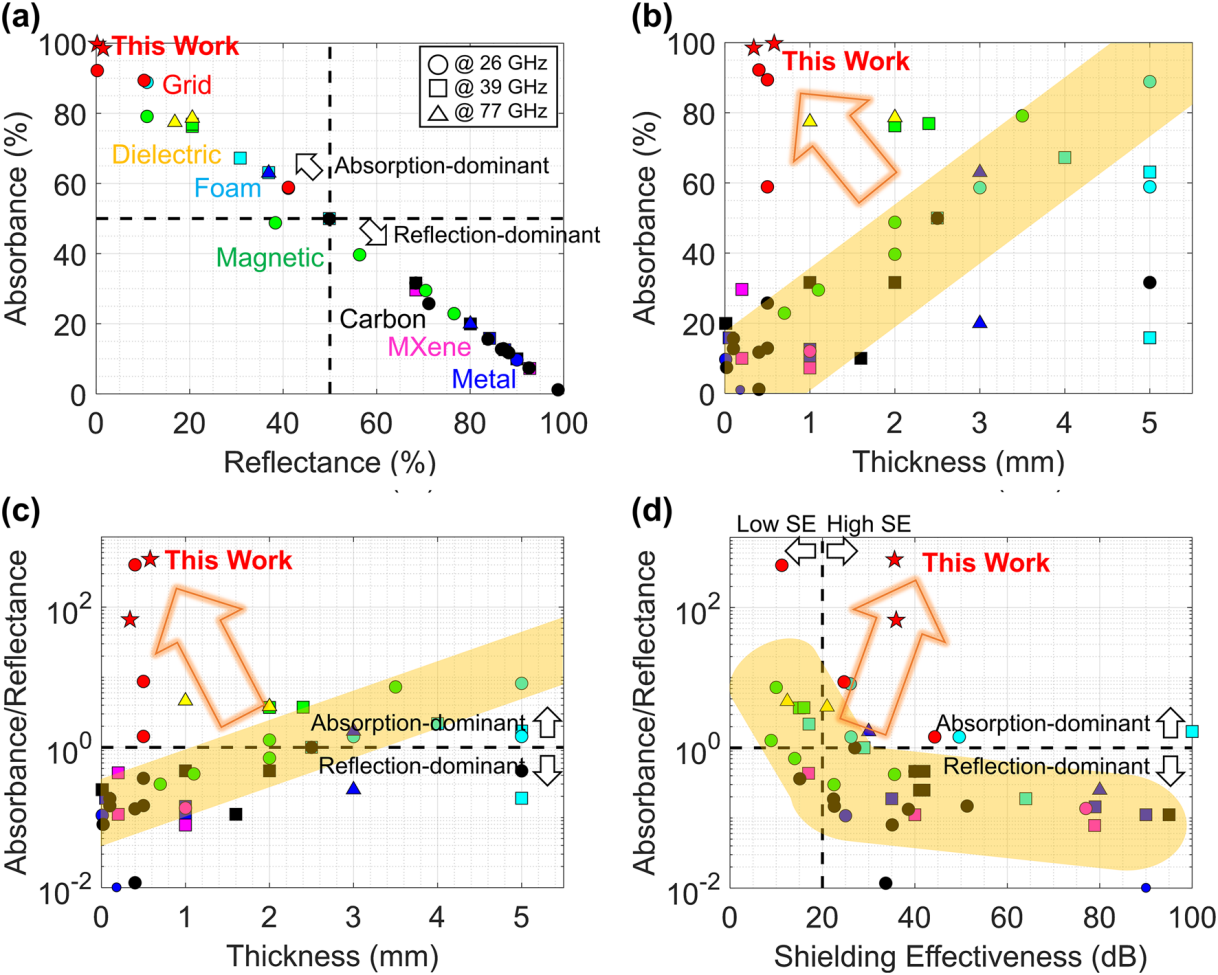
Comparison of the EMI shielding performance of the proposed works (red stars) with previous literature. **a** Reflectance vs. absorbance. **b** Thickness vs. absorbance. **c** Thickness vs. absorbance/reflectance ratio. **d** Shielding effectiveness vs. absorbance/reflectance ratio

Two examples of mmWave EMI shielding films with ultralow reflection are presented, one for 39/52 GHz 5G telecommunication and the other for 60/77 GHz automotive radars. The EMI shielding performance of these films is compared with previously reported shielding materials for mmWave frequencies, including 26 [5, 6, 8–10, 18, 25–31, 98–100], 39 [2, 6, 7, 13, 18, 25, 31–34, 101–106], and 77 GHz [107, 108] (the exact values are presented in Table S1). In Fig. 11a, conductive shielding materials are located on the bottom-right side, indicating that they are reflection-dominant materials that reflect more than 80% of the incident EMI. Magnetic and dielectric composite materials are more absorption-dominant, but their reflectance is still more than 20%. Foam-type shielding materials also show less reflectance, but their thicknesses are usually more than several millimeters, and their applications in mobile devices are limited. On the other hand, composite shielding materials with conductive grids, including the proposed works, outperform other reports with exceptionally ultralow reflectance < 5%.

This is better represented in Fig. 11b–c with thickness comparison. Most shielding materials tend to be thicker to be more absorption-dominant (high absorbance and high absorbance/reflectance ratio (ARR)), as presented with yellow regions in the figures. The proposed shielding films absorb more than 95% of the incident EMI with sub-millimeter thicknesses using FMR tunable doped SrMs for mmWave frequencies and tailored conductive grid design. This helps reduce EMI reflection and minimize its secondary pollution, which is necessary for mmWave applications.

In addition, it should be noted that the proposed films show not only high absorbance but also high SE. Figure 11d shows that shielding materials with high SE tend to be reflection-dominant, while absorption-dominant materials show low SE. As many 5G applications require severe EMI shielding standards higher than 20 dB SE [109, 110], it is important to simultaneously achieve minimized reflectance and maximized SE. To the best of the authors’ knowledge, the proposed films are the only materials performing more than 30 dB SE and 60 ARR, which correspond to more than 99.9% EMI shielding and 98% absorption, and less than 2% EMI reflection. As doped SrM powders in the composite layer effectively absorb electromagnetic energy with its high magnetic loss, it is possible to achieve a high SE while still being absorption dominant.

## Conclusions

In this study, EMI shielding films with multi-band ultralow reflection are proposed. By combining a magnetic composite layer and a conductive grid, an absorption-dominant shielding material with high SE is achieved with sub-millimeter thicknesses. By tuning the (1) FMR frequency of SrM powders in the magnetic composite layer and (2) composite layer geometries, the frequency bands with ultralow reflections can be controlled. Two examples are presented in this study. The first 0.58 mm film shields 34.5–58.5 GHz frequency band using Co-Ti doped SrM powders with an FMR near 39 GHz, which minimizes EMI reflection at 39 and 52 GHz 5G telecommunication frequencies. The other 0.34 mm film shields 53.8–91.8 GHz frequency band using Al doped SrM powders with FMR near 60 GHz, which minimizes EMI reflection at 60 and 77 GHz automotive radar frequencies.

The EMI shielding performance of the proposed films is compared with those of previous literature, which shows that the proposed films outperform other shielding materials with their high absorbance, low reflectance, high SE and sub-millimeter thicknesses. This is necessary for 5G EMI shielding materials to avoid secondary EMI pollution with tight component-to-component spacing in integrated mobile modules. Additional studies are underway to (1) enhance the SE of the films using doped M-type ferrites with higher magnetic loss and (2) achieve additional ultralow reflection bands using complex grid patterns and layer compositions.

### Supplementary Information

Below is the link to the electronic supplementary material.

Supplementary file1 (PDF 577 KB)
