# Supplementary material for: Absorption-Dominant mmWave EMI Shielding Films with Ultralow Reflection using Ferromagnetic Resonance Frequency Tunable M-Type Ferrites
Source: Nanomicro Lett. 2023 Mar 28;15:76. doi: 10.1007/s40820-023-01058-w (PMC10050308; doi:10.1007/s40820-023-01058-w)
Supplement: Supplementary file 1 — Supplementary file1 (PDF 577 KB) [file 40820_2023_1058_MOESM1_ESM.pdf]

Supporting Information for

Absorption-Dominant mmWave EMI Shielding Films with Ultralow Reflection using Ferromagnetic Resonance Frequency Tunable M-Type Ferrites

Horim Lee[1], Seung Han Ryu[1], Suk Jin Kwon[1], Jae Ryung Choi[1], Sang-bok Lee[1], and Byeongjin Park[1,] *

[1]Composites Research Division, Korea Institute of Materials Science, 797 Changwondaero, Seongsan-gu, Changwon, Gyeongsangnam-do 51508, Republic of Korea

*Corresponding author. E-mail: b.park@kims.re.kr (Byeongjin Park)

Supplementary Figures

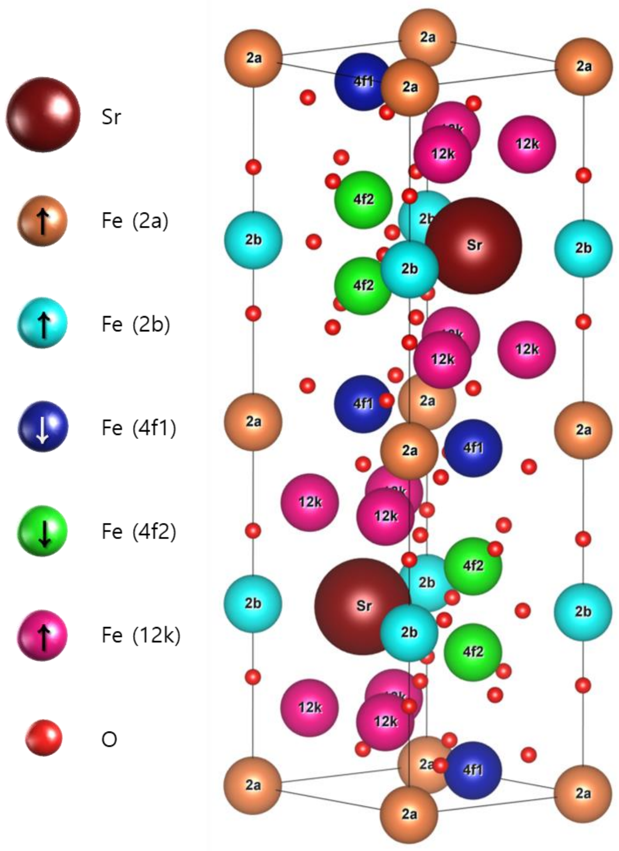

Fig. S1 Crystallographic structure of M-type Strontium ferrite using VESTA software [a]

Figure S1 shows crystallographic structure of M-type strontium ferrite (SrM), containing five different Fe sites: (1) octahedral 2a site with up-spin, (2) trigonal bipyramidal 2b site with up-spin, (3) tetrahedral 4f1 site with down-spin, (4) octahedral 4f2 site with down-spin and (5) octahedral 12k site with up-spin. Due to the antiparallel arrangement of spins, the M_S and H_a of SrM are affected in different ways depending on which site the localized Fe^{3+} ions occupy.

[a] K. Momma, F. Izumi, *Journal of Applied Crystallography* **2011,** *44*, 1272.

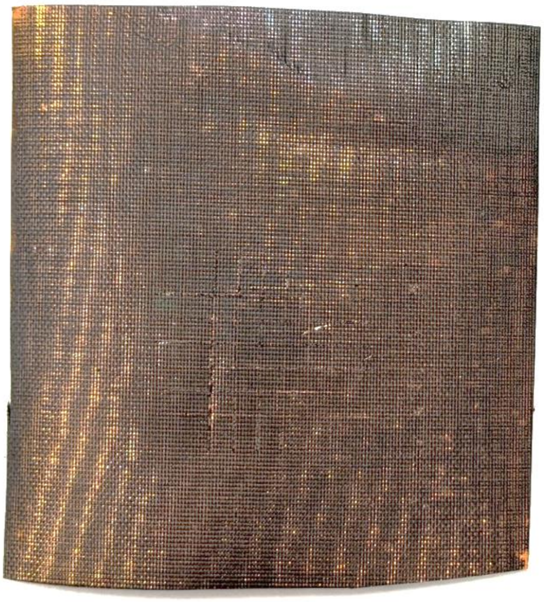

Fig. **S2** ASTM D3359B cross-cut adhesion test on the proposed EMI shielding film [b]

ASTM D3359 Method B is one of the most widely used test method to rate adhesion between a substrate (the composite layer in our paper) and a thin coating film (the Cu grid in our paper). The test procedure is as follows:

1) Make six vertical cuts using a razor blade, and make additional six horizontal cuts centered on the original cuts

2) By applying and removing pressure-sensitive tape over cuts made in the film, the adhesion is evaluated according to the removed area of the film.

As presented in the figure above, almost no Cu grid was removed from the composite layer interface, less than 5% of the total area. This corresponds to 4B/5B classification, which meets the standards of practical industrial applications. Please find the following figure in ASTM standard D3359 to classify adhesion test results.

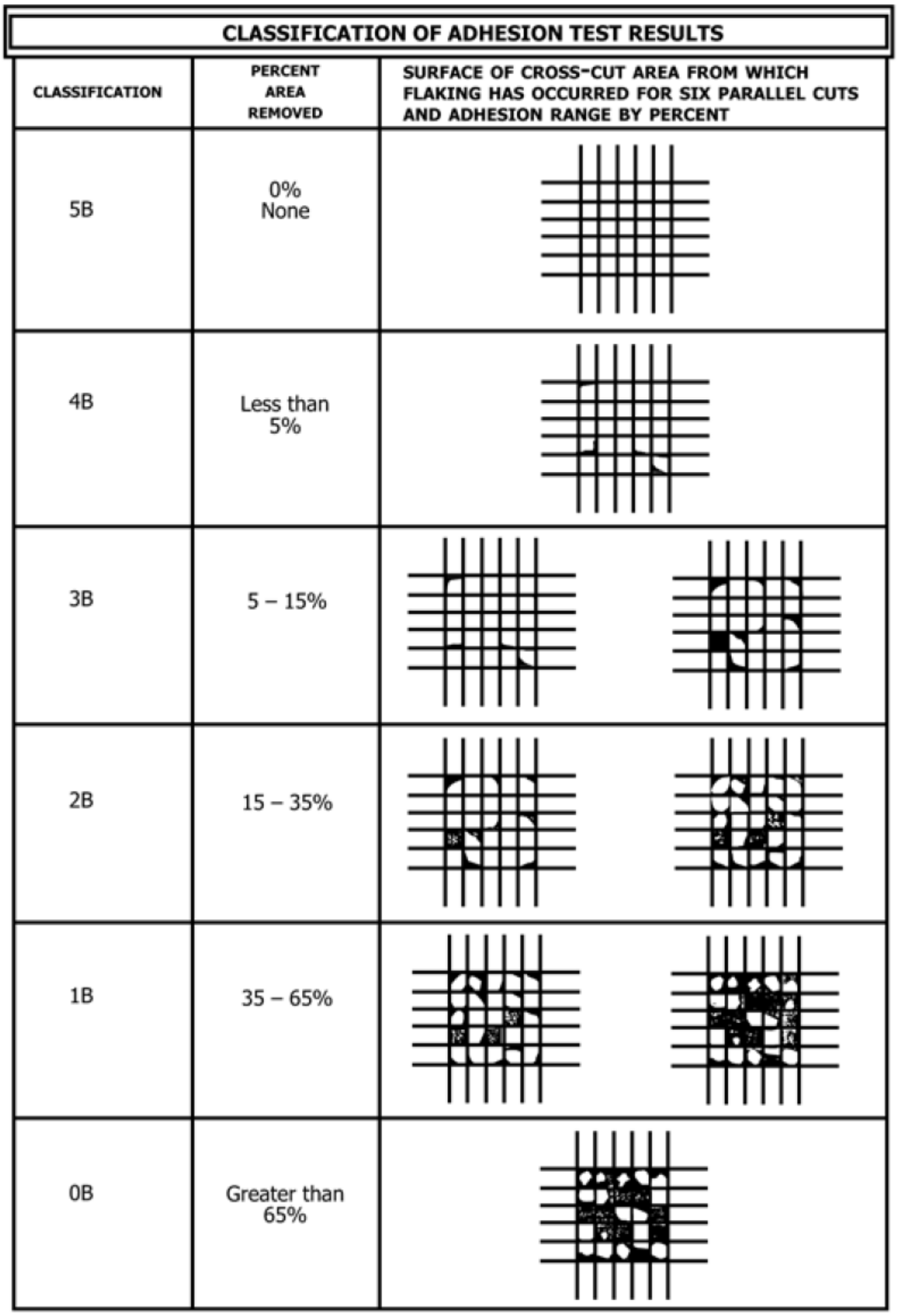

CLASSIFICATION OF ADHESION TEST RESULTS		
CLASSIFICATION	PERCENT AREA REMOVED	SURFACE OF CROSS-CUT AREA FROM WHICH FLAKING HAS OCCURRED FOR SIX PARALLEL CUTS AND ADHESION RANGE BY PERCENT
5B	0% None	
4B	Less than 5%	
3B	5 – 15%	
2B	15 – 35%	
1B	35 – 65%	
0B	Greater than 65%	

FIG. 2 Classification of Adhesion Test Results for Test Method B

[b] ASTM Standard D3395, 2022, “Standard Test Methods for Rating Adhesion by Tape Test,” ASTM International.

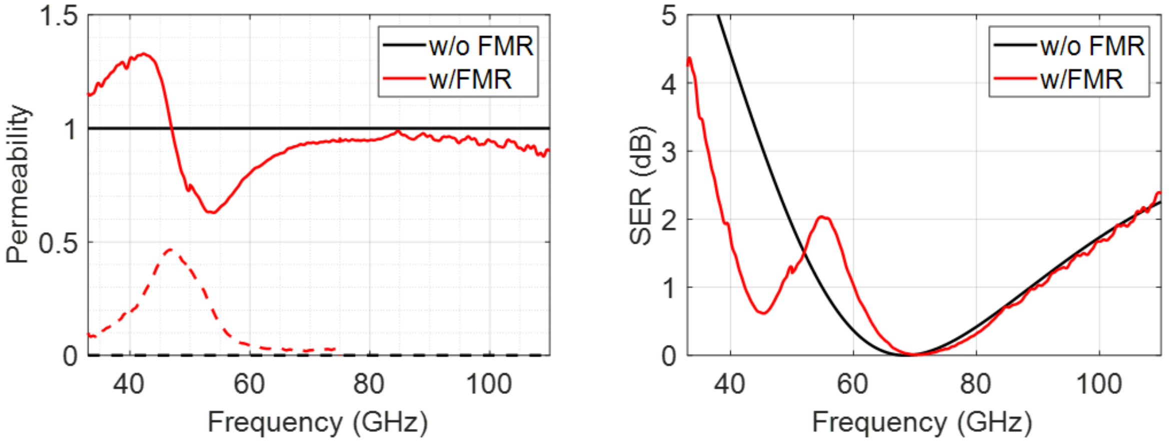

Fig. S3 FMR leads to additional impedance matching and two local minima of SER occurs

The right figure presents the calculated SER for two composites with a same constant permittivity (ε’=8, ε’’=3) and thickness (0.4 mm) but with different permeability (the left figure). Without FMR (black), there is a single local minimum of SER due to its frequency-independent permeability. However, with FMR at 50 GHz (red), the SER decreases around the FMR frequency and additional local minimum occurs, leading two multi-band low reflection.

Table S1 EMI shielding performance of previously reported shielding materials for mmWave frequencies

Form	Main Materials	Target Frequency (GHz)	Thickness (mm)	SER (dB)	SEA (dB)	SE (dB)	R (%)	A (%)	T (T)	ARR (%)	References
Film	Metal	26	0.18	20	70	90	99.0	1.0	100	0.01	[5]
Film	Metal	26	0.01	10	15	25	90.0	9.7	99.7	0.11	[6]
Film	MXene	26	1	9.2	67.8	77	88.0	12.0	100	0.14	[18]
Film	Graphene	26	0.02	11.3	23.8	35.1	92.6	7.4	100	0.08	[98]
Composite	CNT	26	0.5	5.4	9.7	15.1	71.2	25.8	96.9	0.36	[8]
Composite	CNT	26	0.5	8.9	42.4	51.3	87.1	12.9	100	0.15	[8]
Composite	CNT	26	0.1	7.91	14.5	22.4	83.8	15.6	99.4	0.19	[9]
Composite	CNT	26	0.4	9.3	29.3	38.6	88.3	11.7	100	0.13	[10]
Composite	Graphene	26	0.1	8.76	13.8	22.6	86.7	12.7	99.4	0.15	[9]
Composite	Graphene	26	0.4	19.2	14.5	33.7	98.8	1.2	100	0.01	[10]
Composite	Graphene	26	2.5	3	24	27	49.9	49.9	99.8	1.00	[25]
Composite	Graphite	26	5	5	35	40	68.4	31.6	100	0.46	[97]
Composite	Magnetic	26	2	3.6	10.4	14	56.3	39.7	96.0	0.70	[31]
Composite	Magnetic	26	2	2.1	6.8	8.9	38.3	48.8	87.1	1.27	[31]
Composite	Ferrite	26	3.5	0.5	9.5	10	10.9	79.1	90.0	7.28	[26]
Composite	Ferrite	26	0.7	6.3	16.2	22.5	76.6	22.9	99.4	0.30	[99]
Composite	Ferrite	26	1.1	5.3	30.3	35.6	70.5	29.5	100	0.42	[99]
Foam	CNT	26	5	0.5	25.5	26	10.9	88.9	99.7	8.17	[27]
Foam	CNT	26	3	2.3	23.9	26.2	41.1	58.6	99.8	1.43	[28]
Foam	CNT	26	5	2.3	47.3	49.6	41.1	58.9	100	1.43	[28]
Composite	Grid	26	0.4	0.01	11.2	11.2	0.2	92.2	92.4	400.89	[29]
Composite	Grid	26	0.5	0.47	24.2	24.7	10.3	89.4	99.7	8.72	[29]
Composite	Grid	26	0.5	2.3	42	44.3	41.1	58.9	100	1.43	[30]

Film	Metal	39	0.05	8	27	35	84.2	15.8	100	0.19	[6]
Film	Graphene	39	0.008	7	35	42	80.0	19.9	100	0.25	[13]
Film	CNT	39	0.012	7	34	41	80.0	19.9	100	0.25	[102]
Microsphere	Metal	39	1	10	80	90	90.0	10.0	100	0.11	[7]
Microsphere	Metal	39	5	2	98	100	36.9	63.1	100	1.71	[33]
Composite	Metal	39	1	9	70	79	87.4	12.6	100	0.14	[2]
Composite	CF	39	2.4	1	15	16	20.6	76.9	97.5	3.74	[32]
Composite	CNT	39	2	5	35	40	68.4	31.6	100	0.46	[100]
Composite	Graphite	39	1.6	10	85	95	90.0	10.0	100	0.11	[104]
Composite	CNT	39	1	5	37	42	68.4	31.6	100	0.46	[105]
Composite	Carbon	77	3	7	73	80	80.0	20.0	100	0.25	[106]
Composite	Carbon	77	3	2	28	30	36.9	63.0	99.9	1.71	[106]
Composite	MXene	39	1	11.4	67.5	78.9	92.8	7.2	100	0.08	[18]
Composite	MXene	39	0.2	5	12	17	68.4	29.6	98.0	0.43	[103]
Composite	MXene	39	0.2	10	30	40	90.0	10.0	100	0.11	[103]
Composite	Magnetic	39	2	1	14	15	20.6	76.3	96.8	3.71	[31]
Foam	Graphene	39	2.5	3	26	29	49.9	50.0	99.9	1.00	[25]
Foam	CNT	39	4	1.6	15.5	17.1	30.8	67.2	98.1	2.18	[34]
Foam	Metal	39	5	8	56	64	84.2	15.8	100	0.19	[102]
Composite	GF	77	1	0.8	11.6	12.4	16.8	77.4	94.2	4.60	[107]
Composite	GF	77	2	1	20	21	20.6	78.6	99.2	3.82	[107]
**Composite**	**Grid**	**39**	**0.58**	**0.01**	**35.6**	**35.6**	**0.2**	**99.8**	**100**	**481.92**	**This Work**
**Composite**	**Grid**	**60**	**0.34**	**0.07**	**35.9**	**36.0**	**1.5**	**98.5**	**100**	**66.30**	**This Work**
